# Comparing global and community indicators of safety and peace

**DOI:** 10.4102/ajopa.v8i0.200

**Published:** 2026-06-24

**Authors:** Naiema Taliep, Ghouwa Ismail, Shahnaaz Suffla, Mohamed Seedat, Lu-Anne Swart, Ashley van Niekerk, Shrikant I. Bangdiwala

**Affiliations:** 1 Institute for Social and Health Sciences, College of Human Sciences, University of South Africa, Western Cape, South Africa; 2 Violence, Injury and Social Asymmetries Research Unit, South African Medical Research Council, Cape Town, South Africa; 3 Institute for Social and Health Sciences, College of Human Sciences, University of South Africa, Lenasia, South Africa; 4 Population Health Research Institute, Department of Health Research Methods, McMaster University, Ontario, Canada

**Keywords:** safety, peace, global indicators, dimensions, community indicators

## Abstract

Indicators measuring the progress of safety and peace have been pursued at a macro (global) and meso level (community). While global indicators focus on the country-level indicators, it does not consider the community-level contextually relevant needs of the indicators. While the global indicators present a general framework to measure safety and peace, these constructs are dynamic and fluid, and thus understood differently within and across countries, provinces and communities. Drawing on data collected through community participatory processes, this study aims to compare existing global indicators with community-level indicators of peace and safety and to evaluate the usefulness of these global indicators for measuring peace and safety at the community level. This study forms part of a larger study the Community Safety And Peace Index (CSPI) that aimed to develop a contextually and psychometrically valid and reliable instrument to assess, monitor and promote safety and peace at the community level.

**Contribution:**

The analysis of the data revealed several dimensions of safety and peace, including physical (dis)order, social (dis)order, community cohesion, structural security, social justice, affective and interpersonal relationships, crime and violence and values.

## Introduction

Injuries and violence, directly and indirectly, affect the health and well-being of individuals in both the short and long term (University of Wisconsin Population Health Institute, 2015). Recognising that living in unsafe communities can induce trauma and fear of crime and thwart the development of trust and safe interpersonal relationships (Miliauskas et al., 2022), a body of literature has emerged to focus on delineating the dimensions and indicators of safety and peace. Essential to tackling the root causes of violence and measuring progress towards peace is the availability of contextually relevant indicators (De Almagro, 2021) and systems of measurement for gauging safety and peace at the community level. Indicators are vital for promoting change, increasing awareness of existing challenges and gaps and stimulating the promotion of safety and peace (Brusset et al., 2022).

Indicators measuring progress towards safety and peace have been pursued at both the macro (global) and meso (community) levels. While the macro-level indicators present a general framework for measuring safety and peace, the argument is that peace and safety are dynamic phenomena that are understood differently within and across countries, provinces and communities (Brusset et al., 2022). Policymakers and practitioners struggle to define and delineate such abstract concepts, resorting to top-down approaches to measure peace and safety (Churchman, 2022; Lindsay et al., 2021). In making a case for localised indicators, Lindsay et al. (2021) argue that macro-level, universal indicators may not take into account local political, cultural and historical contextual complexities and particularities.

Several shortcomings of global indicator measuring approaches have been noted. Firstly, traditional global approaches have been criticised for not accurately measuring the concepts of interests, but rather, focusing on indicators of the concept identified by a select few (Mac Ginty, 2013a, 2013b, 2013c). These indicators of justice and safety often neglect the perspectives of community members, who are rarely systematically consulted regarding the metrics used to establish the success or failure of safety and peace initiatives (Brett et al., 2024). A significant gap also exists between how local people and external experts perceive indicators of safety and peace (Mac Ginty, 2013c). Hence, global approaches to indicator development have the potential to disenfranchise the very population they intend to empower (Mac Ginty, 2013a, 2013b).

Secondly, traditional indicator systems are limited in terms of range and tend to overlook local political, cultural and historical contexts; hence, they do not account for across or within-group differences that may exist (Lindsay et al., 2021; Mac Ginty, 2013a, 2013b). In addition, Slocum et al. (2024) argue that community safety and peace indicators are relatively underdeveloped worldwide, including practical local community knowledge on these indicators; hence, existing indicators may be at best deficient. Globally, indicators are required to tackle context-specific challenges and priorities at the community level (Slocum et al., 2024).

The need exists for the development of contextually relevant indicators through researcher–community collaboration and the involvement of multiple local stakeholders to obtain a more comprehensive understanding of these concepts and relevant measurement indicators (Kaholokula et al., 2024; Eds. Minkler & Wakimoto, 2022).

Community-informed safety and peace indicator approaches pave the way towards overcoming conventional limitations of macro indicator systems (Kendhammer & Chandler, 2021; Mac Ginty, 2013a; Rich et al., 2021). Community indicators are data that can be combined to provide an overview of what is currently happening in a community or system, encompassing an entire system or community through a concise collection of elements (Rich et al., 2021). These approaches are based on locally produced knowledge and have the potential to better reflect local meaning and context than previously mentioned traditional approaches (Kendhammer & Chandler, 2021; Mac Ginty, 2013a; Rich et al., 2021).

A combination of indicators provides a platform for measuring, assessing, monitoring and evaluating specific phenomena in a community, including directional trends and progress made (Agarwal et al., 2019; Talmage et al., 2021; Valentin & Spangenberg, 2000). This study’s aims are twofold: (1) to review global-level indicators of safety and peace and evaluate their usefulness for measuring safety and peace at a community level and (2) to report on the participatory development of community-level safety and peace indicators, drawing on data from multiple countries in Africa.

## Research methods and design

This research is situated within the realm of psychometric measurement and validity theory, focusing on crafting a contextually valid and reliable measurement tool for assessing and monitoring community safety and peace. This study employed a community-based participatory approach to identify indicators of safety and peace at the community level. This approach signifies a collaborative approach in which academics and community stakeholders jointly identify, describe and validate indicators. The participatory community engagement strategy takes a constructive approach to tackle community issues and concerns, operating within an ecological framework (Taliep et al., 2023). To ensure the contextual relevance and central involvement of community members in the indicator development process, we followed Innes and Booher’s (2000, p. 117) recommendations, which include: (1) ensuring a participatory indicator development process with direct inputs from end-users, (2) measuring something that is publicly valued, (3) ensuring the meaning of these indicators is understood and common among those for whom they are relevant, (4) ensuring indicators are considered credible by experts, (5) ensuring all parties trust the indicators and (6) ensuring indicators have theoretical and practical applicability.

**FIGURE 1:**
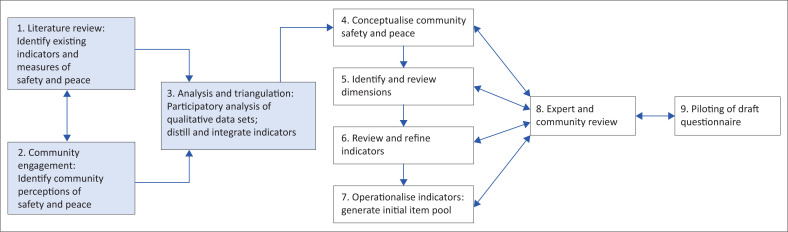
Subphases in the development process of safety and peace indicators.

The development process of the Community Safety and Peace Index (CSPI) is illustrated in Figure 1 and consists of the following steps: (1) conducting a literature review, (2) engaging with communities, (3) analysing and triangulating data, (4) conceptualising community safety and peace, (5) identifying and reviewing dimensions, (6) reviewing and refining indicators, (7) operationalising indicators, (8) conducting expert and community reviews and (9) piloting the draft questionnaire. For this study, we will focus particularly on the first three recommendations proposed by Innes and Booher (2000), which align with the first three phases (see Figure 1) in the participatory development of the CSPI.

As depicted in Figure 1, the development of the CSPI comprises various sub-phases, including initial groundwork for conceptualising safety and peace, as well as identifying safety and peace dimensions and indicators, prior to the development and refinement of the questionnaire items and the piloting of the questionnaire. Here we present steps 1–3; a subsequent article dealt with steps 4–9 (see Taliep et al., 2025). Note that expert and community reviews are part of every development sub-phase.

### Identifying community-level indicators

In this study, our objective was to distil community-level indicators of safety and peace from two participatory datasets, namely the Photovoice project and the SCRATCHMAPS (Spiritual capacities and Religious Assets for Transforming Community Health by Mobilising Males for Peace and Safety) study. Both datasets were particularly well suited to this aim because they employ participatory, community-engaged methodologies that draw on grounded theory and grounded analytical principles to generate insights directly from community members’ lived experiences. Grounded, inductive approaches of this nature are central to developing contextually relevant, culturally valid and ecologically grounded measurement indicators, which strengthen the content validity and real-world applicability of emerging instruments (Charmaz, 2014; Innes & Booher, 2000; Taliep et al., 2025).

#### A multicountry Photovoice project on safety and danger indicators

The primary aim of this project was to explore and elicit young people’s representations of safety in their communities, as characterised by both assets and risks, and stimulate youth-driven safety promotion action.

Key objectives of the study were (1) to enable youth to record and reflect on their representations of safety through small and large group discussions of participants’ sense of agency and activism concerning safety promotion and (2) to develop community-level indicators of safety and danger. These were elicited through a Photovoice methodology pursued with youth in six African countries (South Africa, Mozambique, Uganda, Zambia, Egypt and Ethiopia) around the theme: *Things, places, and people that make me feel safe* and *unsafe in my community* (Suffla et al., 2012). Photovoice is a community-centred approach to conducting research, often, but not always, implemented in marginalised communities (McMorrow & Musoke, 2023). Photovoice methodology has been described as:

A process by which people can identify, represent, and enhance their community through a specific photographic technique. It entrusts cameras to the hands of people to enable them to act as recorders, and potential catalysts for social action and change, in their own communities. It uses the immediacy of the visual image and accompanying stories to furnish evidence and promote an effective, participatory means of sharing expertise to create healthful public policy. (Wang & Burris, 1997, p. 369)

#### The SCRATCHMAPS study

The broad aim of the SCRATCHMAPS study was to explore how the mobilisation of community assets can promote safety and peace by mobilising males to transform community health (Lazarus et al., 2017; Taliep et al., 2023). A key objective of the study was to elicit community members’ views on safety and peace using multiple qualitative research methods to distil community-level indicators for the development of a survey instrument that measures safety and peace at a community level (see Taliep et al., 2025). Community views were generated in response to the question: ‘What would you put in place to ensure a peaceful and safe community?’ Data collection incorporated various innovative participatory methodologies, including: (1) constructing a house of peace and safety using cardboard ‘bricks’ on which community members wrote one factor that would contribute to a peaceful and safe community, (2) teacher-guided learner drawings accompanied by brief written explanations of what a safe and peaceful community would look like and (3) community asset mapping workshops with community members and service providers to identify factors that promote or undermine safety and peace. The multiple data sources generated through these methods were subsequently triangulated to extract core dimensions and indicators of safety and peace.

This triangulated, community-driven process ensured that the indicators reflected both lived experiences and the structural conditions shaping everyday safety and peace. The Photovoice dataset provided youth-driven, experiential, visual and narrative accounts of everyday safety and danger, while the SCRATCHMAPS dataset contributed broader community-wide, intergenerational and structural perspectives through its multimethod data collection processes. Together, these two grounded, participatory approaches offered complementary lenses that strengthened methodological triangulation and ensured that the resulting indicators reflected a multidimensional, contextually grounded understanding of community safety and peace.

### Sampling and participant recruitment

The Photovoice study was conducted in low-income communities in South Africa, Uganda, Mozambique, Zambia, Egypt and Ethiopia. All participants were actively attending school during the study period and were recruited through both formal and informal youth organisations within their respective communities. Utilising established partnerships with community stakeholders aided the recruitment process. Participant identification was facilitated by community leaders associated with local non-governmental and community-based organisations operating within the communities. Additionally, community-engaged researchers and scholars in other countries assisted in identifying existing participants. A total number of 85 participants were recruited for the study, with gender distribution as follows: in Cape Town, South Africa, 50% (*n* = 10) were female and 50% (*n* = 10) were male; in Maputo, Mozambique, 50% (*n* = 5) were female and 50% (*n* = 5) were male; in Kampala, Uganda, 50% (*n* = 5) were female and 50% (*n* = 5) were male; in Lusaka, Zambia, 50% (*n* = 5) were female and 50% (*n* = 5) were male; in Ismailia, Egypt, 36% (*n* = 10) were female and 64% (*n* = 16) were male and in Axium, Ethiopia, 50% (*n* = 8) were female and 50% (*n* = 8) were male (Suffla et al., 2015). The age of participants ranged from 11 years to 17 years (Suffla et al., 2015).

While there were slightly more males among the Photovoice study participants, the majority of SCRATCHMAPS participants were female. Three Community Asset Mapping (CAM) workshops were conducted in a low-income community comprising around 250 households in the Western Cape, South Africa. These workshops attracted 74 attendees aged 18 years and above from diverse faith backgrounds (Christianity, Islam, Khoisan and Rastafarianism), facilitated by wide-reaching invitations through posters, flyers and door-to-door invitations.

The Service Provider Mapping (SPM) workshop was attended by 18 service providers from various sectors.

Two action planning workshops brought together 63 local participants, including stakeholders and community members, with a nearly equal split between sexes (34 males, 29 females). To complete the ‘bricks’, local participants’ age groups, ranging from 13 years to 85 years, were conveniently sampled. The bricks were completed by 175 participants, with a slightly lower number of males (*n* = 69) than females. Of the respondents who completed the learner essays, most participants identified as female (*n* = 44; 60.27%) and were all Grade 7 primary school children, aged 12–14 years, from two local schools in the community.

### Data analysis

Data analysis for the Photovoice study employed qualitative visual discourse analysis (McMorrow & Musoke, 2023), which involves analysing the photographs, participants’ accompanying narratives and group discussions to identify key themes and insights related to safety and danger from the perspective of young people.

All datasets from the SCRATCHMAPS study were analysed using a participatory *a priori* thematic framework analysis method (Taliep, 2016; Taliep et al., 2023). The initial predetermined categories for the analysis comprised: (1) community members’ meanings or understandings of the key concepts (safety, peace), (2) factors that promote safety and peace and (3) factors that hinder safety and peace. The predetermined subcategories, utilised under each of the main categories, included: (1) tangible factors, (2) intangible factors and (3) systems levels (individual, relationship, community and societal). These levels aligns with Bronfenbrenner’s Ecological System’s framework, which captures the multilayered influences shaping community safety and peace, which posits that individuals’ experiences of safety are embedded within and shaped by multiple, interrelated ecological levels, from the immediate microsystem of family, peers and local neighbours, to the broader exosystem and macrosystem contexts encompassing social structures, institutional practices and cultural norms (Bronfenbrenner, 1979). To facilitate the collective and participatory process of data analysis, academics within the research team provided training in qualitative thematic analysis to the community research team, which consisted of 10 local community members. The community research team was subdivided into groups and undertook the analysis of the datasets. These were then summarised in two tables, that is, what the community would like to see, and what they want to do to build a safe and peaceful community. An academic then validated the data analysis.

**FIGURE 2:**
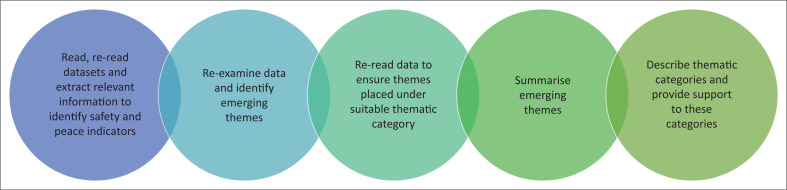
Data analysis procedure.

The data analysis process for the learner essays and the asset mapping data consisted of five steps, as outlined in Figure 2.

These categories, as shown in Figure 2, provided a framework for the development of the indicators. All three datasets were analysed according to an identical initial analysis framework, wherein understandings of safety and peace were drawn out and where factors that promote safety and factors that negate safety and peace were identified. The subsequent steps in the analysis were identical for the learner essays and asset mapping report but differed when the ‘bricks’ for the house of peace and safety were examined. The bricks were divided into two groups: those that represented ‘unsafe/unpeaceful’ factors. Similar bricks were grouped, and the frequency of each idea was determined by adding up the total number of ‘bricks’ within each main group. Following this, the predetermined subcategories outlined above (i.e. tangible, intangible and systems-level factors) were used to group the ‘bricks’ according to their subcategories and emerging themes.

### Ethical considerations

Ethical clearance for the study was obtained from the University of South Africa Human Science Ethics Committee (CHS-CREC), University of South Africa (NHREC Registration No: Rec-240816-052; CREC Reference No: 2019-CHS-CREC – 0273). Ethical clearance and written informed consent and assent had been previously obtained from the Multicountry Photovoice Project on Safety and Danger Indicators and the SCRATCHMAPS Study to further distil and identify indicators of peace and safety from these two studies.

## Results and discussion

The integrated results and discussion are presented in two parts: firstly, the global-level indicators reviewed in subphase 1 and secondly, the community-level dimensions and indicators identified through subphase 2.

### Subphase 1: Reviewing the existing body of knowledge – Macro-level indicators of safety and peace

This section outlines the global-level indicators of safety and peace and explains how they informed the early conceptual framing for the development of community-level indicators. The primary steps in developing a measuring instrument involve reviewing the literature to establish whether similar instruments already exist and to provide a clear conceptualisation of the constructs that underpin the measurement instrument (i.e. the CSPI). In this paper, we focus specifically on global measures of safety and peace. Table 1 outlines the dimensions and indicators used to measure macro-level indices.

**TABLE 1:** Global measures and indicators of safety and peace.

Global peace index	Positive peace index	National culture of peace	Measuring progress in conflict environment	Human development index	Governance, peace, and security index	South African development indicators
Ranks countries’ level of peacefulness through three domains: **Ongoing domestic and political peace** (# internal / external conflict; # deaths from organised conflict; level of organised conflict; relations with neighbouring countries) **Societal safety and security** (perception of criminality in society; political instability; # homicides; level of violent crime; # jailed population) **Militarisation** (military expenditure as % of GDP; # armed service personnel per 100 000 population; access to small arms/light weapons) IEP (2017; 2023a)	Measures eight domains, each with three indicators that comprise Positive Peace: **Well-functioning government** (openness & transparency; effectiveness; rule of law) **Sound business environment** (regulatory quality; financial institutions index; GDP per capita) **Low levels of corruption** (control of corruption; factionalised elites; public sector theft) **High levels of human capital** (youth not in employment, education, training; researchers in R&D; Healthy life expectancy) **Free flow of information** (press freedom; quality of information; % population using internet) **Good relations with neighbours** (law on equal treatment; international tourism; external intervention) **Equitable distribution of resources** (inequality-adjusted life expectancy index; access to public services; equality of opportunity) **Acceptance of the rights of others** (gender inequality; group grievance; exclusion by socio-economic group) IEP (2023b)	Measures quality of life through six domains: **Physical and mental health** (life expectancy and employment rate) **‘Brotherhood’** (social security and child abuse deaths) **Peace** (deaths in foreign wars) **Order** (homicides and deaths in civil strife) **‘Variety’** (press freedom scores) **Progress** (per capita gross national product; per capita contributions to science) Razafindrakoto and Roubaud (2018).	MPICE measures the drivers of violent conflict vis-à-vis ability to resolve the conflict peacefully. Using five end states essential to resolving conflict: **Safe and secure environment** (e.g. diminished political violence, threat from ex-combatants; and strengthened compliance with security agreements, performance of national security forces, and public confidence in security forces, etc.) **Political moderation and stable governance** (e.g. diminished political grievances, and external destabilisation; and strengthened peace process, services, and free responsible media, etc.) **Rule of law** (e.g. diminished injustice, impunity, and criminalisation of state institutions; and strengthened public order and safety, and administration of justice, etc. **Sustainable economy** (e.g. diminished economic inequality between groups, and effects of economic decline; and strengthened infrastructure, fiscal integrity, and financial institutions, etc.) **Social well-being** (e.g. diminished population displacement and societal cleavage; and strengthened peace process and access to basic needs, etc.) Agloglia et al. (Eds. 2010)	Measures a country’s average achievements in three domains of human development: **A long and healthy life** (life expectancy at birth) **Knowledge** (years of schooling and expected years of schooling) **Decent standard of living** (GNI per capita in PPP terms in US$) Klugman et al. (2011)	Measures three dimensions: **Human rights and participation** (civil and political rights; participation; absence of discrimination and gender inequality) **The rule of law** (judicial system; absence of corruption) **Peace and Security** (national security; public safety) Adechian (2020)	Contains 10 dimensions and 92 individual indicators: **Economic growth and transformation** (e.g. Gross Domestic Product (GDP) growth, inflation, interest rates and government debt, etc.) **Employment** (e.g. unemployment, employment, etc.) **Poverty and inequality** (e.g. per capita income, living standard measure, poverty gap analysis, etc.) **Household and community assets** (e.g. sanitation, potable water, electricity, etc.) **Health** (e.g. life expectancy, malaria, tuberculosis, immunisation, etc.) **Education** (e.g. enrolment and literacy rates, and access to quality education, etc.) **Social cohesion** (e.g. voter participation, strength of civil society, etc.). **Safety and security** (e.g. crimes, number of inmates, parole, and probation, etc.) **International relations** (e.g., peace operations, etc.) **Good governance** (e.g. corruption, audits, etc.) Department of Planning, Monitoring and Evaluation (2014)

Note: Please see the full reference list of the article Taliep, N., Ismail, G., Suffla, S., Seedat, M., Swart, L.-A., Van Niekerk, A., & Bangdiwala, S.I. (2026). Comparing global and community indicators of safety and peace. *African Journal of Psychological Assessment, 8*(0), a200. https://doi.org/10.4102/ajopa.v8i0.200, for more information

GDP, gross domestic product; IEP, Institute for Economics and Peace; MPICE, Measuring Progress in Conflict Environments; US$, Unites States dollar; R&D, Research and Development; GNI, Gross National Income; PPP, Purchasing Power Parity.

As depicted in Table 1, global-level safety and peace indicators include the Global Peace Index (Institute for Economics and Peace [IEP], 2017), the Human Development Index (United Nations Development Programme [UNDP], 2016), Measuring Progress in Conflict Environments (Eds. Agloglia et al., 2010) and the South African Development Indicators (Department of Planning, Monitoring and Evaluation, 2014).

The Global Peace Index (GPI) measures global peacefulness using 23 qualitative and quantitative indicators, which were selected and reviewed by a panel of experts. The GPI aims to measure the level of negative peace through three thematic domains: (1) ongoing domestic and international conflict (six indicators), which measures the degree to which countries are embroiled in domestic and external conflicts and their role and length of time involved in conflicts, (2) level of societal safety (11 indicators) assessing peacefulness revealed by low levels of crime, terrorism and violent demonstrations; a stable political environment, harmonious relations with neighbouring nations and a low number of the population internally displaced or made refugees and (3) extent of militarisation (six indicators) measuring the relation between military build-up and weapon access and a country’s level of peacefulness at both domestic and international level (IEP, 2017). Nasar and Naqvi (2024) explain that even though the focus of the GPI is on negative peace (delineated as ‘the absence of violence or the fear of violence’), the instrument transcends the analysis of ‘armed conflict’ and centres on violence more generally. It provides an important shift in the narrative and analysis from ‘conflict’ related to a ‘peace’ index. Nair (2016) highlights the following critique levelled at the GPI: the availability of data for each indicator in different countries, the combination of elements that do not point in the same direction (e.g. ‘military expenditure’ might not necessarily increase conflict), the absence of ‘violence against women’ as an indicator and the weight given to the components of the Index that leads to an imbalanced ranking of countries. For example, Equatorial Guinea (a repressive dictatorship, which censors and crushes protests) is ranked 81, whereas South Africa was ranked 136 out of 162 countries, ranking the country as substantially less peaceful than Equatorial Guinea.

Recently, the IEP developed the Positive Peace Index (PPI) to supplement the GPI. Positive Peace is defined as the ‘attitudes, institutions and structures that create and sustain peaceful societies’ (IEP, 2018, p. 6). The IEP has identified eight domains, or Pillars, that comprise positive peace: a well-functioning government, a sound business environment, recognition of the rights of others, low levels of corruption, high levels of human capital, a free flow of information, good relations with neighbours and an equitable distribution of resources. The PPI can be utilised to ‘measure a country’s resilience – its ability to absorb, adapt and recover from shocks, such as climate change or economic transformation’, help calculate the probability of conflict, violence and instability and indicate areas where policy intervention could contribute to peace (IEP, 2018, p. 7; Syropoulos et al., 2021). A key drawback of both the GPI and the PPI is that they largely neglect the cultural and subjective domains of peace that influence people’s viewpoints (Das, 2024; Nasar & Naqvi, 2024).

The Human Development Index (HDI) measures a country’s overall social and economic development using three dimensions: a long and healthy life, level of educational attainment and standard of living or gross national income per capita (UNDP, 2016). The HDI is based on international data from the United Nations Population Division, the United Nations Educational, Scientific and Cultural Organisation Institute for Statistics and the World Bank (UNDP, 2016). Key drawbacks of the HDI include its being a simplification and limited evaluation of human development, and it does not consider the quality-of-life aspects, such as overall feelings of security (Yin et al., 2023).

Measuring Progress in Conflict Environments (MPICE, pronounced M-Peace) is a metric framework of indicators that aim to identify potential sources of ongoing violent conflict and instability at a national level and to longitudinally measure progress in reducing the means and precursors of violent conflict and enhancing citizens ability to resolve conflict peacefully using five domains, namely governance, sustainable economics, safety and security, rule of law and social well-being (Eds. Agloglia et al., 2010; Khosa & Abdulkareem, 2023).

The MPICE guide recommends a range of quantitative and qualitative data collection and analysis methods that can be used in conjunction with the MPICE framework, including content analysis, expert knowledge, quantitative data and survey data (Eds. Agloglia et al., 2010; Khosa & Abdulkareem, 2023). Increasing government institutional performance and reducing conflict drivers are key to increasing stability, as outlined in MPICE, which essentially serves as a monitoring and evaluation tool within the stabilisation context (Stabilization Unit, 2014). The MPICE inventory consists of several stabilisation outcome measurements, arranged into five essential ‘sectors’ that are necessary for conflict resolution: (1) safe and secure environment, (2) political moderation and stable governance, (3) rule of law, (4) sustainable economy and (5) social well-being (Eds. Agloglia et al., 2010). Like the GPI and the HDI, the MPICE comprises universal indicators developed by experts using top-down approaches. Apgar et al. (2024) caution that universal indicators may not adequately account for local political, cultural and historical contextual complexities and argue that local indicators are better suited to address unique local circumstances (Apgar et al., 2024).

The Governance Peace and Security Index (GPSI) measures perceptions of population groups at a sub-regional level and utilises the methodology adopted to compute the Global Governance Index (Adechian, 2020). The GPSI measures three dimensions, namely ‘human rights and participation’, ‘rule of law’ and ‘peace and security’ (Adechian, 2020, p. S27). The demand for data in Africa generally focuses on conventional macroeconomic management areas and sectoral programmes, but there has been an increase in recent years in requests for data related to new policy and development areas, including human rights and freedom, governance, and democracy. This requirement is addressed through GPI macro-level indicators by applying data from the eight West African Economic and Monetary Union member states’ Integrated Regional Survey on Employment and the Informal Sector (Adechian, 2020).

The South African Development Indicators (SADI) serve as quantitative benchmarks to monitor progress towards key policy objectives in the country. Tracking these indicators allows for the evaluation of South Africa’s developmental path, pinpointing areas that require enhancement and crafting targeted interventions to promote sustainable development and inclusive growth. The SADI comprises 10 dimensions and 92 individual indicators: (1) Economic Growth and Transformation (16 indicators); (2) Employment (4 indicators); (3) Poverty and Inequality (7 indicators); (4) Household and Community Assets (6 indicators); (5) Health (9 indicators); (6) Education (10 indicators); (7) Social Cohesion (9 indicators); (8) Safety and Security (13 indicators); (9) International Relations (5 indicators) and (10) Good Governance (7 indicators) (DPME, 2014). Data are gathered from various sources, including official statistics (Statistics South Africa), government administrative systems (South African Reserve Bank), national census data and research from international institutions (DPME, 2014). Key challenges associated with the SADI encompass concerns regarding data reliability and consistency across the various sources utilised, which stem from disparities in reporting standards and methodologies (Statistics South Africa, 2018). There are further constraints regarding data accessibility and availability, notably in marginalised and remote regions, and the dynamic nature of development challenges necessitates ongoing enhancements in methodologies to accurately capture the intricate dynamics of development (Statistics South Africa, 2018).

Global indicators of peace and safety, as illustrated in Table 1, provide a comprehensive overview of peace and safety worldwide, focusing on key factors such as political stability, governance, international relations, conflict resolution and military expenditures. Compiled by international organisations, these indicators draw data from diverse sources, including government reports, surveys and expert assessments. While global indicators offer a broad perspective on peace and safety trends at a macro level, community-level indicators provide insights into the lived experiences of individuals within specific communities. Unlike macro-level indicators that focus on more high-level and aggregate data across global populations, often comprising data on militarisation, international wars, terrorism or the overall level of violence in countries, community-level indicators can provide data on multiple levels, from individual personal and community-specific data to larger structural factors that hamper safety and peace.

Several attempts have been made to develop community-informed indicators of safety and peace. Such measures include the Everyday Peace Indicator (Mac Ginty, 2013a, 2013b) and the Community Resilience Measure (Ahmed et al., 2004). The Everyday Peace Indicator (EPI) used an inductive or bottom-up approach to identify community-informed indicators of peace and social change, particularly in societies emerging from violent conflict, and uses a participatory framework to design and develop these indicators (Mac Ginty, 2013a, 2013b). The EPI was developed to assess subjective and locally pertinent indicators of peace in societies recovering from conflict, especially those transitioning from civil war or political violence (Mac Ginty & Firchow, 2016, Firchow & Mac Ginty, 2017). The dimensions of the EPI, which focuses to a large degree on peace-building and positive peace, include: (1) security, (2) dealing with the past, (3) rights and dignity (these three dimensions are the most state-oriented, addressing state-guaranteed rights, transitional justice mechanisms, and institutions such as the military and police), (4) armed actors, (5) culture and safety and (6) livelihood and health (Firchow & Dixon, 2025). The Community Resilience Measure (CRM) offers another example of a community-participatory framework used to identify community-relevant indicators. Like the EPI, the CRM questionnaire was developed through several participatory steps involving community members. The dimensions of resilience were operationalised, delineating the following seven measures of community resilience: business ownership, household security, communal unity, community structures, social support, access to knowledge and community hope (Ahmed et al., 2004). The CMR was used to measure resilience in three local neighbourhoods in the Western Cape (Ahmed et al., 2004).

The next subsection describes how community-level indicators were extrapolated using a grounded analytical approach applied to the datasets.

### Subphase 2: Distilling indicators and dimensions using a grounded analytical approach

The Photovoice and SCRATCHMAPS datasets were analysed separately using grounded analytical principles, and their findings were later triangulated to generate a unified set of community-level indicators.

Although global indicators of safety and peace provide a broad overview of country-level data, given their shortcomings, identifying community-level indicators was deemed crucial for assessing everyday safety and peace at the community or neighbourhood level. This study used a grounded analytical approach to identify community-level indicators of peace and safety. This method comprises a systematic and iterative participatory process that involves the community, gathers data and analyses it systematically. Themes and concepts arose directly from the lived experiences and viewpoints of community members who possess valuable knowledge and insights regarding safety and peace challenges within their community.

As outlined below, subphase 2 of the study comprised distilling the core indicators of peace and safety from the Photovoice and SCRATCHMAPS projects.

#### A multicountry photovoice project on safety and danger indicators

The core dimensions and indicators elicited through the Photovoice study using young people’s visual images and their accompanying stories are outlined in Table 2. Categories or measures for both safety and danger emerging from this study included: (1) Physical Order/Physical Disorder, (2) Social Order/Social Disorder, (3) Security/Lack of Security and (4) Affective and Interpersonal/Relational Dimension.

**TABLE 2:** Dimensions and indicators extracted from the photovoice study.

Dimension	Safety indicators	Dimension	Danger indicators
**Physical order**	Community safety and security infrastructure Maintained physical environment Access to basic infrastructure	**Physical disorder**	Littering Dilapidated physical structures Poorly maintained facilities Exposure to sources of burns Lack of traffic infrastructure Absence of facilities
**Social order**	Prosocial community behaviours and actions Presence and visibility of law enforcement	**Social disorder**	Child and female labour Public drinking Unsupervised children Presence of gangs
**Security**	Access to food, water Economic security Access to shelter Access to education Health and access to health facilities	**Lack of security**	Inadequate food Inadequate water/wastage Poverty, unemployment Inadequate health facilities Absence of shelter Lack of/inadequate access to education
**Affective and interpersonal/relational dimension**	Family Friends/peers Larger group support Sense of security Protection by others	**Affective and interpersonal/relational dimension**	Poor relationship with family Poor relationship with friends/peers Poor relationship with a larger support group Poor sense of connectedness Lack of emotional security Risk of victimisation by others

The above views from community members provide a useful example and baseline for developing indicators at the community level. It is important to note that, unlike the global indices, this study highlighted both positive (safety) and negative (danger) factors. To complement the youth-centred insights from Photovoice, SCRATCHMAPS provided broader community-level, intergenerational perspectives.

#### The SCRATCHMAPS study

The core dimensions and indicators that emerged from the SCRATCHMAPS study, categorised by tangible and intangible factors at each level of Bronfenbrenner’s ecological system, are outlined in Table 3.

**TABLE 3:** Dimensions and indicators extracted from the SCRATCHMAPS study.

Systems level	Dimensions	Intangible	Tangible
Individual	Social order (pro-social and safety values, behaviours and actions)	**Values** Respect; compassion; love Positive values/morals; positive mindset; hope/vision Love and care for others Emotional freedom Honesty **Actions and behaviours** Taking responsibility Caring for animals Trust Prayer and/or meditation A positive sense of identity Personal growth; healing	Dress nicely and cleanly
Relationship	Social order Cohesion/connectedness Social justice Social support	**Family** Family unity and cohesion Family socialisation (rules and obedience) Healing and building families **Other relationships** Trust; working and standing together	**Supervision/guidance** Adult guidance and supervision Peer guidance **Domestic violence** Absence of women and child abuse Absence of domestic violence
Community	Structural security Community cohesion/connectedness Physical order Social order Culture of safety	**Community cohesion and unity** Working/standing together Neighbourhood watch working with the community Positive values Police working with the community Trust No corruption Healing/positivity Responsibility in community	**Activities** Sports, youth, recreational activities Community projects NPO/NGO activities **Safe environment** Community and street clean Facilities; community hall Trees and lighting **Government/community services/opportunities** Employment Housing Non-formal education Strong community leaders Community committee **Decrease in criminal activity** Drug-free Crime and violence-free No gambling spots **Safe places** Churches Safe parks and streets Safe spaces for children to play
Societal level	Structural security		**Government services and service delivery** Employment/job opportunities Improved quality and access to formal education Access to drug rehab and education Housing Access to social development services Access to healthcare

Table 3 summarises what emerged from the triangulation process. Participants associated peace and safety with both tangible (e.g. the presence of safe spaces) and intangible factors (e.g. values such as compassion, respect and care).

The core dimensions that emerged from both the SCRATHMAPS and Photovoice studies included physical (dis)order, social (dis)order, structural security, social justice, affective and interpersonal relationships, community cohesion or connectedness, crime and violence and values. These were identified as additional dimensions in the SCRATHMAPS study. Although the dimensions identified in the two studies are largely similar, there were noteworthy differences in the indicators identified by participants across the two studies to measure these dimensions. In the SCRATCHMAPS study, for example, honesty, care (for animals, the environment and others), trust, compassion, respect, love, prayer and meditation emerged as important indicators of social order. Whereas in the Photovoice study, indicators of social order included the presence and visibility of law enforcement, reduced child and female labour, public drinking, gangs and unsupervised children, alongside prosocial behaviours and actions.

Together, the two participatory datasets enabled the identification of eight core dimensions of community safety and peace. These convergent dimensions, drawn from diverse age groups and social positions, laid the groundwork for the subsequent phases of indicator refinement and operationalisation.

### Limitations

One significant drawback of this study is that we only identified community-level dimensions and indicators within low-income African settings. While these indicators might apply to other low-income settings worldwide, the community-driven approaches we adopted could be considered by others in various settings; however, different cultural and social contexts may yield different indicators.

## Conclusion

This study directly addresses the 2030 Agenda for Sustainable Development imperative to reduce all forms of violence, build peaceful, just and inclusive societies, measure the drivers of peace and work with communities. To achieve this goal, reliable and contextually valid safety and peace indicators and measures are imperative. The participatory development of community-level safety and peace indicators using a grounded analytical approach represents an important endeavour to produce measures that are contextually valid and reliable at the community level. Hence, the primary focus of this study was to address the need for the development of contextually relevant indicators of safety and peace using community-based participatory research methods.

The identification of community-level indicators of safety and peace has several important implications. These indicators offer a more contextually grounded and culturally meaningful basis for assessing everyday safety and peace than global, top-down indices, which often overlook local lived realities and socio-historical contexts. By reflecting tangible and intangible elements (including physical and social disorder, structural conditions, interpersonal dynamics and community values), the indicators provide a multidimensional framework that can be used to inform the design, monitoring and adaptation of community-based interventions. They also offer policymakers, practitioners and local stakeholders a tool for identifying priority areas for change, tracking progress over time and strengthening context-sensitive programming. Moreover, because the indicators are derived from community members’ experiences and language, they enhance the likelihood of local uptake, legitimacy and sustainability of safety and peace initiatives.

A further strength of this study is the integration of visual and community-based participatory methodologies (i.e. Photovoice and SCRATCHMAPS), which yielded a richer, more nuanced set of indicators than either method could have produced alone. The Photovoice process allowed young people to document and interpret everyday experiences of safety and danger using visual imagery, narrative reflections and group dialogue, providing access to experiential, emotional and place-based understandings that are often difficult to capture through traditional methods. SCRATCHMAPS, on the other hand, elicited intergenerational and structural insights through participatory workshops, asset mapping, community-generated ‘bricks’ and learner reflections, highlighting broader community dynamics, collective aspirations and social-ecological factors shaping safety and peace.

Together, these methods reveal complementary layers of meaning: Photovoice captures micro-level lived experiences and sense-making, while SCRATCHMAPS surfaces community-level processes, assets and challenges. Their combined use strengthens triangulation, enhances the depth and validity of the indicators and ensures that both subjective and structural dimensions of safety and peace are represented. Importantly, both approaches draw on grounded analytical principles, allowing indicators to emerge inductively from the data rather than being imposed externally. This participatory, grounded approach strengthens the ecological and cultural validity of the resulting measurement framework and aligns with best practice in indicator development, which emphasises the need for locally meaningful constructs that resonate with communities’ lived realities and priorities (see Ismail & Van Niekerk, 2021; Taliep et al., 2014).

Our findings show that, while macro-level (global) indicators present a general framework for measuring safety and peace, these measures were developed by a select few using top-down approaches and do not accurately reflect community members’ conceptions of what safety and peace constitute. These constructs are not fixed or static; instead, they change and adapt over time and are shaped by context. These concepts are interpreted and perceived differently depending on the specific geographical and social context, such as at the national level (across different countries), the regional level (across provinces or states) and the local level (across various communities). Global indicators typically target measurable outcomes rather than underlying issues. This raises doubts about the development of globally applicable indicators that can be applied equally to all contexts, particularly in diverse community settings. Cultural, social and environmental factors may be overlooked by peace and security indicators because they prioritise certain dimensions over others.

While macro-level indicators, undoubtedly, provide a helpful broader perspective on safety and peace that encompasses a wide range of contexts, community-level indicators focus on specific localities, regions, districts, towns or local communities and often assess safety and peace at the grassroots level, which is important for addressing safety, peace and other public health challenges that often vary by community, frequently alongside other geo-political, structural and socio-economic challenges.

Community-level indicators are crucial for aligning intervention research with community needs and for establishing the effectiveness and sustainability of initiatives.

Firchow and Mac Ginty’s (2017) Everyday Peace Indicators framework highlights local voices in peace assessments; however, it is best suited for post-conflict settings rather than for everyday community violence marked by chronic structural inequality and state neglect, which requires both subjective perceptions of safety and peace as well as objective indicators of structural conditions and experiences of unsafety.

By using a grounded analytical approach within a community-based participatory framework, this study ensured that the indicators were deeply rooted in the lived experiences and perspectives of community members, thereby enhancing the relevance of the indicators. Community-level indicators were distilled from two primary studies, that is, the SCRATCHMAPS and Photovoice studies. Eight key dimensions, comprising 94 indicators, emerged from the studies. The dimensions included: physical (dis)order, social (dis)order, community cohesion, structural security, social justice, affective and interpersonal relationships, crime and violence and values. While the dimensions are similar across both studies, there are differences in participant-identified indicators for measurement. In terms of social order, for example, the Photovoice study had six indicators, whereas the SCRATCHMAPS study identified 17 indicators.

The next steps will comprise conceptualising community safety and peace, identifying and reviewing dimensions, reviewing and refining indicators, operationalising indicators and generating the initial item pool (i.e. all the items that were distilled from the data), expert and community review employing face-to-face consensus building using concurrent verbalisation with verbal probing and lastly, piloting the draft instrument (see Taliep et al., 2025).
